# Presentation of Superior Vena Cava Syndrome as a Consequence of Fibrosing Mediastinitis

**DOI:** 10.7759/cureus.101085

**Published:** 2026-01-08

**Authors:** Kaitlyn Unterman, Christopher Gamard, Amelia Dorr, Kathleen Clark

**Affiliations:** 1 Internal Medicine, Alabama College of Osteopathic Medicine, Dothan, USA; 2 Internal Medicine, Mobile Infirmary Medical Center, Mobile, USA

**Keywords:** endovascular stenting, fibrosing mediastinitis, histoplasmosis, superior vena cava (svc) syndrome, suspicious lung mass

## Abstract

Fibrosing mediastinitis (FM) is a rare inflammatory reaction within the mediastinum that causes diffuse fibrosis and can lead to compression of vascular structures in the thoracic and cervical regions. While typically benign and indolent, significant progression can lead to obstruction of vital organs, causing conditions like superior vena cava (SVC) syndrome, pulmonary hypertension, and potentially fatal right-sided heart failure. The clinical presentation often mimics malignancy, which necessitates exclusion. FM is a rare condition, with idiopathic cases being even rarer. While histoplasmosis or tuberculosis infections are common causes of FM in the US, idiopathic forms such as IgG4-related FM can also occur.

## Introduction

Fibrosing mediastinitis, also known as sclerosing mediastinitis, can be described as a progressive inflammatory reaction to either an infectious, non-infectious, malignant, or idiopathic etiology that leads to fibrous replacement of the mediastinum. Although FM is usually a diagnosis of exclusion, it can be traced back to multiple origins. The exact etiology is not always easy to ascertain, but the most common trigger in North America is thought to be *Histoplasma capsulatum* [1]. Mycobacterial infections, as well as noninfectious origins such as sarcoidosis, systemic lupus erythematosus, and IgG4-related disease, have also been implicated [2]. FM mimics malignancy by forming dense, fibrous masses that radiologically look like tumors on CT and PET scans. Both malignancy and FM can present with irregular borders and infiltrative masses that can cause secondary lung issues such as atelectasis or nodules. These commonalities represent the difficulty of diagnosis, necessitating a biopsy. However, the degree of calcification on radiography is highly characteristic. Malignancies rarely demonstrate a high level of calcification. The timeline of diagnosis is what makes investigating the etiology unclear. Most patients are diagnosed on incidental findings due to the continual progression of fibrosing processes leading to superior vena cava (SVC) syndrome, such as in this patient. Prior to compression due to the SVC syndrome, most patients remain asymptomatic; therefore, determining the inciting infection or etiology can be difficult.

The superior vena cava is the largest systemic vein of the mediastinum, formed by the junction of the right and left brachiocephalic veins, and is responsible for carrying blood from the head, neck, and upper extremity back to the heart [3]. Partial or complete obstruction of this blood flow leads to SVC syndrome. Vascular blockage causes an increase in venous pressure in the thoracic region, leading to facial plethora or erythema along with distended jugular veins, cough, and dyspnea [4]. While the most common causes of SVC syndrome are small cell bronchogenic tumor infiltration of the vascular walls or non-Hodgkin lymphoma, other benign etiologies making up about 40% of cases, include thrombus formation due to semipermanent intravascular catheters or pacemaker wires, as well as mediastinitis due to a fibrosing process [4].

We present a case report of a 56-year-old male patient experiencing SVC syndrome secondary to fibrosing mediastinitis. FM is a rare but significant condition characterized by the proliferation of diffuse fibrosis within the mediastinum, potentially compressing vascular structures. This case is noteworthy due to the rarity of FM, especially when idiopathic, and highlights the diagnostic challenge when symptoms mimic malignancy. Early recognition and a multidisciplinary approach are crucial for managing symptoms and improving outcomes in SVC syndrome.

## Case presentation

**Figure 1:**
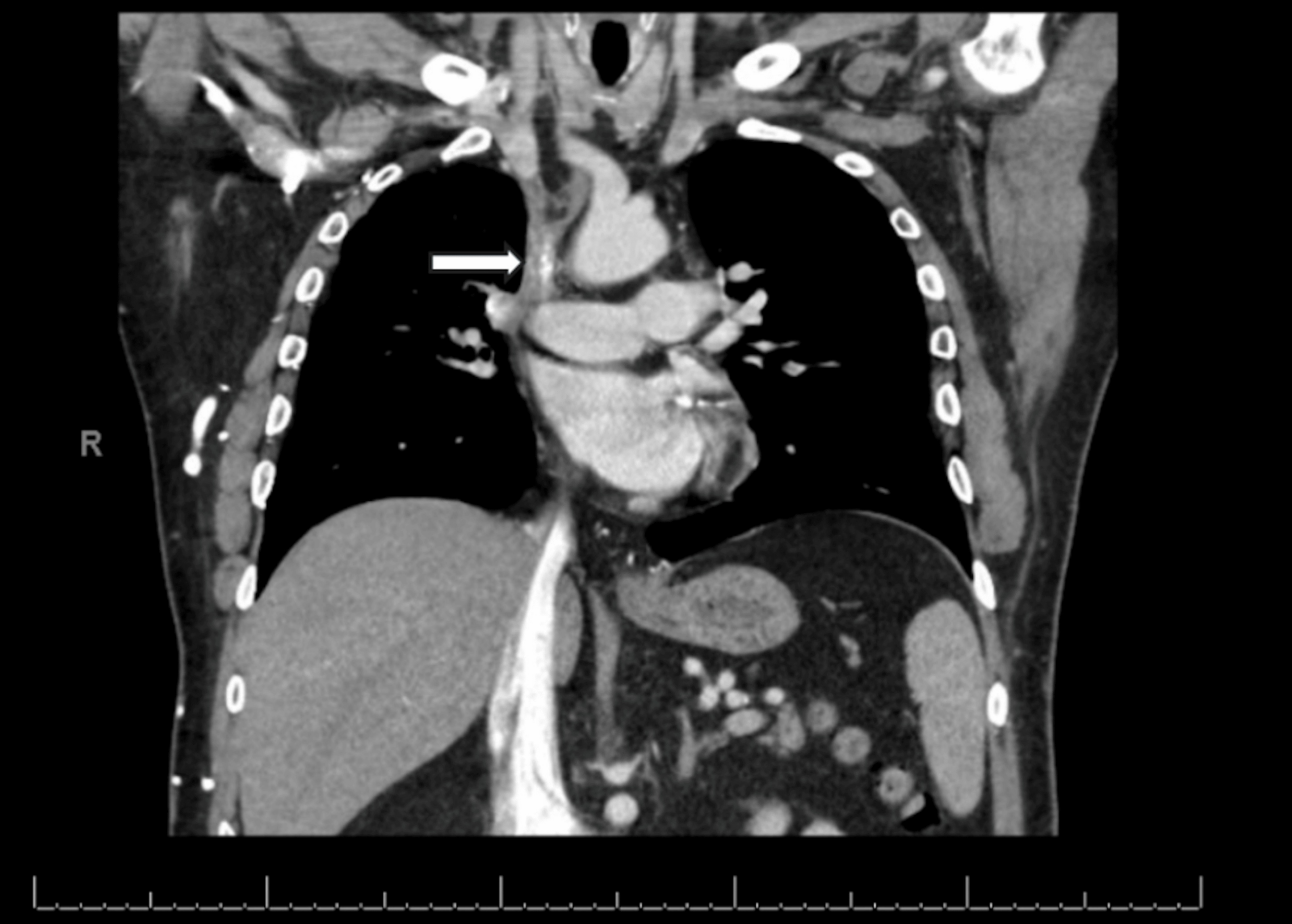
Superior vena cava stenosis and calcification secondary to external pressure from fibrosing mediastinitis. Contrast-enhanced computed tomography of the chest. Coronal view.

A 54-year-old male patient who is 6'1" and 256 pounds with past medical history including diabetes mellitus, hypertension, coronary artery disease, hyperlipidemia, and insomnia, presented with dizziness, headache, and dyspnea on exertion. He was a former smoker who quit 10 years prior with a 2.7 pack-year history. His past employment was in international shipbuilding. The patient reported gradually worsening syncopal episodes when bending over or with heavy exertion, such as mowing the lawn in the heat. This was associated with swelling of the hands and arms, facial plethora, distended superficial thoracic veins, and occasional right arm numbness. They were consistent with a diagnosis of SVC syndrome, which was confirmed through computed tomography of the chest (Figure 1).

**Figure 2:**
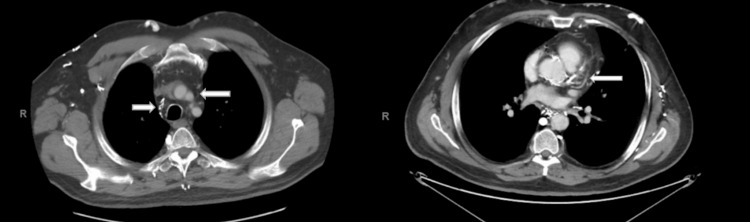
Fibrosing mediastinitis in a 56-year-old male; diffuse infiltrative mediastinal tissue (indicated by arrows) with calcifications. Contrast-enhanced computed tomography of the chest. Axial view.

The patient was initially getting worked up for back surgery and was found to have an abnormal EKG and echocardiogram, prompting further investigation. While undergoing workup for coronary artery disease, imaging showed lung and mediastinal abnormalities. A contrast-enhanced computed tomography of the chest demonstrated a diffuse infiltrative process involving the mediastinal tissue (Figure 2).

Bronchoscopy was then performed, which resulted in massive hemoptysis of 750 mL, and the patient subsequently underwent bronchial artery embolization. Diagnosis of fibrosing mediastinitis was then made based on CT imaging in conjunction with the clinical symptoms, and bronchogenic malignancy was ruled out following the biopsy. The patient was referred to vascular surgery, and an intravascular ultrasound confirmed bilateral innominate vein occlusions. Ultimately, the patient was referred to a tertiary care center where he underwent a covered stent placement in the right and left innominate veins as well as angioplasty and stenting of the SVC. Completion venous imaging revealed excellent flow through both sides without collateral filling and brisk filling into the right atrium. He was discharged on Eliquis and aspirin and planned to be followed with venous duplex bilateral upper extremity imaging. He continues to be followed by vascular surgery, pulmonology, cardiology, and internal medicine, and is scheduled to have a CT scan with contrast three months post-stent procedure.

## Discussion

This case underscores the challenges in diagnosing fibrosing mediastinitis, particularly due to its rarity and the tendency to mimic malignancy. Malignancy can mimic FM, often requiring biopsy to rule out a malignant disease process [5]. FM can follow an indolent course, with symptoms only appearing after significant progression. A combination of clinical signs, symptoms, and imaging, coupled with consultations with cardiology, pulmonology, and internal medicine, contributed to the diagnosis of FM with complicated SVC syndrome. This patient showed hallmark signs of SVC syndrome, including edema of the face, neck, and upper extremities due to high venous pressure caused by the blockage of the mediastinal vessels due to diffuse fibrosis. Insomnia and headache with associated dizziness were likely due to the increased intracranial pressure [6]. Most notably, the patient experienced bendopnea, or dyspnea within 30 seconds of bending while not holding one’s breath, highly suggestive of SVC obstruction [7]. Ultimately, this patient’s timeline highlights the silent progression of fibrosing mediastinitis, with presentation often appearing after significant vascular compression.

This patient's occupation in international shipbuilding was considered a possible risk factor in the disease’s development and possible exposure to *Histoplasma capsulatum*, which may have been encountered in shipyards or during international travel. Occupational exposure as an international shipbuilder is especially significant because it emphasizes the need for a thorough evaluation to rule out a malignant mediastinal process such as mesothelioma over a fibrosing process [8]. Most cases of FM are thought to be an immune-mediated hypersensitivity response to prior histoplasmosis, often presenting years after the primary exposure, which seemed to be the case in this patient [9]. While histoplasmosis and tuberculosis are common etiologies of FM in the US [10,11], it is important to acknowledge the existence of idiopathic forms, including IgG4-related FM. Prior to surgical intervention, the patient underwent a course of itraconazole, which had shown benefit in prior interventions for suspected histoplasmosis exposure [1]. For patients with acute symptoms, an immunocompetent state, a prolonged state of illness, and enlarging hilar or mediastinal adenopathy, such as in this patient, it is recommended in the 2025 guidelines to consider itraconazole treatment [12].

Early recognition and confirmation of this pathophysiological process is done by imaging. CT angiography is crucial for evaluating the degree of vascular involvement [13]. Specifically, CT with IV contrast is necessary because of its ability to delineate the exact location of the vascular lumen. This is particularly important when co-diagnosing SVC syndrome [14]. The immediate outcome in this patient following the bronchial biopsy was significant hemorrhage, leading to an ICU stay and a hospital transfer for embolization. This complication stresses the importance of the availability of surgical interventions such as bronchoscopy, balloon closure, bronchial artery embolization (BAE), or a combination of both in managing hemoptysis in this rare disease [15]. Endovascular treatment has become the mainstay of modern therapy and has become the first line for its high efficacy and low complication rate. The preferred treatment for SVC syndrome is balloon angioplasty with stent placement in the central venous segment, which is especially helpful in patients with benign etiologies [6]. Without treatment, severe pulmonary hypertension and right-sided heart failure, or death can occur [16]. Covered stents, such as the ones used in this patient, use an inert membrane that limits intimal hyperplasia while allowing endothelialization. They are able to trap unstable plaques or thrombus, thus preventing re-embolization [17]. Better stent patency has been shown to be achieved by using dual antiplatelet therapy with aspirin or clopidogrel or a combination of both, as well as anticoagulation therapy with warfarin, enoxaparin, or a factor Xa inhibitor rather than anticoagulation alone [18]. Even with anticoagulation, the majority of patients require repeat angioplasty and stenting 6-12 months after initial interventional stenting [3].

## Conclusions

This case report highlights the rarity of this pathophysiological process as well as the importance of discussing the difficulties in diagnosing this condition. Infiltrative FM can lead to the obstruction of mediastinal structures, including vessels, necessitating a thorough clinical workup and imaging. In addition, the management and treatment of fibrosing mediastinitis are very much defined by the etiology and extent of the disease. With that in mind, patient care can greatly benefit from a multidisciplinary approach with close collaboration of medical and surgical specialists.
